# Multimodal neuro-ophthalmologic changes and the impact of CPAP therapy in severe obstructive sleep apnea: a prospective OCT, OCTA, and visual field study

**DOI:** 10.1007/s10633-026-10122-w

**Published:** 2026-06-11

**Authors:** Yagmur Inalkac Gemici, Uzeyir Ozturk, Mustafa Erdogan, Suleyman Sami Ilker, Hikmet Yilmaz, Aysın Kısabay Ak

**Affiliations:** 1 Department of Neurology, Faculty of Medicine, Manisa Celal Bayar University, Uncubozköy Mah Cella Bayar Ünv., 45030 Yunusemre, Manisa Turkey; 2 Department of Ophthalmology, Faculty of Medicine, Manisa Celal Bayar University, Manisa, Manisa Turkey

**Keywords:** Obstructive sleep apnea syndrome, Continuous positive airway pressure, Optical coherence tomography angiography, Retinal nerve fiber layer, Visual field, Deep capillary plexus

## Abstract

**Purpose:**

To evaluate structural, microvascular, and functional neuro-ophthalmologic changes in severe obstructive sleep apnea syndrome (OSAS) and to investigate the effects of 6-month continuous positive airway pressure (CPAP) therapy using a multimodal approach.

**Methods:**

In this prospective case–control study, 38 patients with PSG-confirmed severe OSAS and 19 controls were enrolled. All participants underwent optical coherence tomography (OCT), optical coherence tomography angiography (OCTA), and visual field (VF) testing (24–2 and 10–2). Thirty patients with adequate CPAP adherence were reassessed after 6 months. Baseline comparisons were additionally adjusted for body mass index (BMI), and false discovery rate (FDR) correction was applied for multiple comparisons.

**Results:**

Baseline neuro-ophthalmologic differences between OSAS patients and controls did not remain statistically significant after BMI adjustment and FDR correction. Following CPAP therapy, significant increases in average and inferior peripapillary retinal nerve fiber layer (RNFL) thickness were observed and remained significant after FDR correction. Significant improvements were also detected in 24–2 VF indices (VFI, MD, and PSD) and 10–2 PSD values after treatment. In contrast, OCTA-derived vascular parameters and macular thickness changes did not remain significant after correction for multiple comparisons. All polysomnographic parameters and Epworth Sleepiness Scale scores improved significantly after treatment.

**Conclusions:**

Although baseline cross-sectional neuro-ophthalmologic differences were attenuated after BMI adjustment and multiple-comparison correction, longitudinal analyses demonstrated significant structural and functional improvement following CPAP therapy in severe OSAS. Multimodal assessment combining OCT and retinal and macular perimetry may provide a useful framework for monitoring treatment-related neuro-ophthalmologic changes in OSAS.

## Introduction

Obstructive Sleep Apnea Syndrome (OSAS) is a disorder characterized by recurrent episodes of upper airway obstruction during sleep, leading to oxygen desaturation and sleep fragmentation [1]. Disease severity is commonly classified according to the apnea–hypopnea index (AHI) obtained from polysomnography (PSG) [2–4]. Continuous positive airway pressure (CPAP) remains the standard treatment for moderate-to-severe disease and has been shown to improve systemic and neurologic consequences of nocturnal hypoxia [5, 6].

In OSAS, recurrent intermittent hypoxia has been shown to affect the optic nerve and retinal structures through mechanisms involving endothelial dysfunction, oxidative stress, and microvascular dysregulation [7, 8]. These pathophysiological processes have been reported to result in structural and functional alterations in various ophthalmologic parameters, particularly in the retinal nerve fiber layer (RNFL) [9, 10].

A substantial body of literature has investigated the association between OSAS and ophthalmologic diseases [11, 12]. These studies emphasize the importance of comparative analyses with appropriate control groups and multimodal imaging approaches in understanding the pathogenesis and clinical manifestations of ophthalmologic involvement across different severities of OSAS. However, to our knowledge, no study has simultaneously evaluated optical coherence tomography (OCT), optical coherence tomography angiography (OCTA), and visual field (VF) parameters before and after treatment in patients with OSAS using a within-subject pre- and post-treatment design.

The present study aimed to investigate whether ophthalmologic alterations observed in OSAS improve following CPAP therapy, to evaluate the potential effects of CPAP treatment on ophthalmologic parameters, and to identify which biomarkers may be most informative for monitoring ocular involvement over time. In particular, the inclusion of both 24–2 and 10–2 visual field strategies together with OCT and OCTA, targeting the assessment of the retina, macula, and ganglion cell layer, may provide a more sensitive multimodal framework for detecting subtle central functional impairment and treatment-related changes in OSAS.

## Materials and methods

### Study design and ethical approval

Ethical approval was obtained from the Institutional Health Sciences Ethics Committee (Decision No: 314; 20.10.2021). This study was supported by the Institutional Scientific Research Projects Coordination Unit (Project No: 2022-024).

This study was designed as an observational, prospective investigation. Between 2022 and 2025, patients evaluated at the Sleep Disorders Clinic of our university who were clinically suspected to have OSAS underwent PSG, and a definitive diagnosis of OSAS was established. Patients with an AHI ≥ 30 who met the eligibility criteria were enrolled after providing written informed consent.

### Inclusion and exclusion criteria

The duration variable represented the patient-reported duration of clinically recognized OSAS-related symptoms before diagnosis, based on information obtained from both patients and their relatives. To obtain a relatively homogeneous cohort and reduce variability related to long-standing disease burden, patients reporting OSAS-related symptoms for longer than one year were excluded. PatiTowith PSG-confirmed severe OSAS who used CPAP therapy regularly (at least 5 days per week and at least 4 h per night) were included. CPAP usage duration was objectively verified using the device memory. Patients using PAP modalities other than CPAP were excluded.

Individuals with a history of chronic pulmonary diseases such as chronic obstructive pulmonary disease or asthma; previous meningitis or encephalitis; neurological disease; congestive heart failure; hypertension; diabetes mellitus; autoimmune disease; systemic vascular disease; malignancy; intellectual disability; or psychiatric disorders that could affect cognitive functions were excluded.

In addition, participants with chronic steroid use, a history of trauma, strabismus, gaze palsy, or any ophthalmologic pathology detected during the examination—including glaucoma, optic neuropathy, non-arteritic anterior and posterior ischemic optic neuropathy, optic disc diseases (e.g., optic disc drusen, optic disc edema/papilledema, or congenital optic disc anomalies), uveitis, retinal vascular diseases (arterial or venous disorders, such as retinal artery/vein occlusion) or anterior/posterior segment pathology—were excluded. Measurements not meeting reliability criteria for OCT, OCTA and VF (false negative > 15%, false positive > 15%, fixation loss > 20%) were excluded. To minimize the potential confounding effect of refractive error on OCT and OCTA measurements, participants with a spherical equivalent outside − 3.00 to + 3.00 diopters were excluded. For OCTA imaging, scans with inadequate image quality were repeated, and eyes with persistently poor-quality images were excluded from the analysis.

### Demographic and clinical data

Demographic characteristics (age and gender), body mass index (BMI), Epworth Sleepiness Scale (ESS) scores, and PSG parameters were recorded. PSG parameters included the apnea–hypopnea index (AHI), mean oxygen saturation (mO_2_), lowest nocturnal oxygen saturation (Lsat), and cumulative time with reduced oxygen saturation (Tsat).

In patients initiated on CPAP therapy, ophthalmologic assessments were repeated only in those whose CPAP use at an effective duration and pressure was confirmed. Pre- and post-treatment comparisons were performed within this subgroup.

### Epworth sleepiness scale

The Epworth Sleepiness Scale was administered to all participants to evaluate excessive daytime sleepiness. The ESS is a self-report instrument that rates the likelihood of falling asleep in eight common daily situations on a scale from 0 to 3, yielding a total score ranging from 0 to 24 [13]. In the present study, the Turkish version with established validity and reliability was used, and the questionnaire was completed during the outpatient visit under standard instructions by the same investigator (ÜÖ) [14].

### Ophthalmologic assessment

All ophthalmologic examinations were performed at the Ophthalmology Outpatient Clinic by the same study investigator (ME). As part of the neuro-ophthalmologic examination, visual acuity, intraocular pressure (IOP) measurement, and fundus examination were performed using the same devices in all participants. Individuals with any ophthalmologic pathology that could meet exclusion criteria were not enrolled.

All participants underwent overnight PSG. The severe OSAS group consisted of treatment-naïve patients diagnosed based on PSG findings, defined by an AHI > 30 events/hour. The control group consisted of healthy volunteers who had undergone PSG for snoring complaints and were confirmed to have normal PSG results. Participants in the control group met the same inclusion and exclusion criteria as the OSAS group and had normal neuro-ophthalmologic examination findings, including normal intraocular pressure (IOP). In addition to routine clinical assessment, OCT, OCTA, and VF examinations were performed in both the OSAS and control groups to evaluate retinal layer thickness and retinal microvascular parameters.

Because this was an observational study, routine clinical management and follow-up of OSAS patients were maintained by the Sleep Disorders Clinic. In patients diagnosed with severe OSAS for whom CPAP therapy was planned, PSG with CPAP titration was performed to determine the appropriate pressure level, and therapy was initiated at the effective pressure that provided marked symptomatic improvement. At the sixth month of CPAP therapy, the same ophthalmologic assessments were repeated. For consistency across multimodal analyses, the same eye was used for OCT, OCTA, and visual field evaluations in each participant. The eye with the worse baseline MD value was selected for statistical analyses.

### Optical coherence tomography (OCT)

Optical coherence tomography examinations were performed using the Stratus OCT device (Carl Zeiss Meditec Inc., Dublin, CA, USA). In the peripapillary region, retinal nerve fiber layer (RNFL) thickness was automatically measured for four quadrants (superior, inferior, nasal, and temporal) as well as the global average RNFL thickness.

For macular assessment, parafoveal thickness (1.5 mm from the fovea) and perifoveal thickness (5 mm from the fovea) were measured in the nasal, temporal, superior, and inferior quadrants. Central foveal thickness, macular volume (mm^3^), and ganglion cell layer (GCL) mean and minimum thickness values were recorded.

### Optical coherence tomography angiography (OCTA)

Optical coherence tomography angiography examinations were performed using the Optovue Inc. device (Fremont, CA, USA). Vascular structures of the retinal and choroidal layers were automatically assessed, including vessel densities of the superficial and deep capillary plexuses, foveal avascular zone (FAZ) parameters, and radial peripapillary capillary network analysis.

Multiple images were obtained from each participant, and analyses were performed over a 3.4-mm diameter circular area centered on the optic disc. Quadrants were defined as superior, inferior, nasal, and temporal.

### Visual field assessment

Visual field examinations were performed using the same perimetry device (Carl Zeiss Meditec, Jena, Germany) by the same technician in all participants. Appropriate near refractive correction was provided during testing, and artificial tears were administered before the test in participants with dry eye.

The perimetry parameters analyzed included mean deviation (MD), pattern standard deviation (PSD), and visual field index (VFI). MD was considered a measure of global sensitivity loss across the visual field. PSD reflected localized irregularities and focal functional loss. VFI, which is closely related to ganglion cell function, represents overall functional integrity of the visual field expressed as a percentage.

The SITA-Fast 24–2 test was performed for retinal evaluation, whereas the 10–2 test was used for macular assessment. In the 24–2 test, a total of 54 points were tested at 6° intervals, while the 10–2 test evaluated 68 test points at 2° intervals around the fovea.

### Statistical analysis

Statistical analyses were performed using IBM SPSS Statistics version 23. The distribution of continuous variables was assessed using the Shapiro–Wilk test. Since the data were normally distributed, comparisons between two independent groups were performed using the independent samples t-test. In addition, analysis of covariance (ANCOVA) adjusted for BMI was performed for baseline patient–control comparisons to evaluate the potential confounding effect of BMI. For pre- and post-treatment comparisons, paired samples t-tests were used. Spearman correlation coefficients were used to evaluate correlations between PSG and demographic parameters and ophthalmologic measures, and to explore interrelationships among OCT structural metrics, OCTA microvascular parameters, and VF indices. Categorical variables were evaluated using the chi-square test or Fisher’s exact test, as appropriate. To reduce the risk of false-positive findings related to multiple comparisons, false discovery rate (FDR) correction using the Benjamini–Hochberg procedure was applied separately within each parameter family.

Results were presented as mean ± standard deviation or median (minimum–maximum) for quantitative variables, and as frequency and percentage for categorical variables. Statistical significance was set at p < 0.05.

## Results

### Participants

Patients with severe OSAS were screened and assessed for eligibility according to the inclusion and exclusion criteria. Initially, 44 patients and 20 healthy controls were enrolled. Due to poor compliance with ophthalmological examinations and/or insufficient test reliability, 6 patients and 1 control were excluded. Therefore, the final analysis included 38 patients with severe OSAS and 19 controls.

### Demographic and clinical characteristics

The mean age was 52.1 ± 10.1 years in the severe OSAS group and 46.7 ± 9.1 years in the control group. The gender distribution was 28/10 (Male/Female) in the patient group and 15/4 in the control group. Body mass index (BMI) was 32.1 ± 5.8 kg/m^2^ in the severe OSAS group and 27.7 ± 0.3 kg/m^2^ in the control group. The mean reported OSAS symptom duration was 6.51 ± 2.09 months. There were no significant differences between the OSAS and control groups in age, sex, educational level, or intraocular pressure (p > 0.05). However, BMI was significantly higher in the severe OSAS group compared with controls (p < 0.001). BMI differed between groups, which is expected in OSAS cohorts; however, its potential confounding impact is likely minimized because the main treatment effects were evaluated using paired pre- and post-CPAP comparisons within the same patients.

### Epworth sleepiness scale

Epworth Sleepiness Scale (ESS) scores were significantly higher in the severe OSAS group than in controls (p < 0.001). After 6 months of continuous positive airway pressure (CPAP) treatment, ESS scores significantly decreased compared with baseline (p = 0.025).

### Polysomnographic parameters

When polysomnographic parameters were compared between groups, the severe OSAS group demonstrated significantly higher AHI and Tsat, as well as significantly lower mO_2_ and Lsat than controls (p < 0.001, p < 0.001, p = 0.005, and p = 0.001, respectively). After adjustment for BMI using ANCOVA, all PSG-related parameters and ESS scores remained statistically significant. Demographic and polysomnographic are summarized in Table 1.

**Table 1:** Comparison of demographic and polysomnographic characteristics between the groups

Parameter	Severe OSAS (n = 38)	Control (n = 19)	p			
Age (years)	52.1 ± 10.1	46.7 ± 9.1	0.427			
Gender (M/F)	28/10	15/4	0.610			
BMI (kg/m^2^)	32.1 ± 5.8	27.7 ± 0.3	** < 0.001**			
Education (P/S/H/U/G)	15/5/7/9/2	5/4/3/5/2	0.458			
OSAS duration	6.51 ± 2.09	N/A				
IOP (mmHg)	23.35 ± 0.78	23.14 ± 0.73	0.816			

PSG parametres			p	Adjusted p ANCOVA for BMI	FDR p	Partial η^2^
ESS	9.6 ± 6.6	0.4 ± 0.3	** < 0.001**	** < 0.001**	**0.005**	.391
AHI	52.3 ± 25.7	2.8 ± 1.2	** < 0.001**	** < 0.001**	**0.002**	.490
Lsat	75.3 ± 9.1	89.2 ± 2.5	** < 0.001**	** < 0.001**	**0.001**	.384
mO_2_	91.2 ± 4.3	94.1 ± 1	**0.005**	**0.029**	**0.029**	.085
Tsat	24.2 ± 23.8	5.9 ± 3,14	** < 0.001**	**0.013**	**0.016**	.109

OSAS, obstructive sleep apnea syndrome; M/F, male/female; BMI, body mass index; P/S/H/U/G, primary/secondary/high school/university/graduate; IOP, intraocular pressure; PSG, polysomnography; ESS, Epworth Sleepiness Scale; AHI, apnea–hypopnea index; mO_2_, mean oxygen saturation; Lsat, lowest oxygen saturation; Tsat, time with low oxygen saturation. Raw p values were obtained using independent-samples Student’s t-tests. Adjusted p values were calculated using ANCOVA with BMI as a covariate. FDR-adjusted p values were calculated using the Benjamini–Hochberg procedure within each parameter family. Partial eta squared values are presented as measures of effect size

### Ophthalmological findings: patient–control comparison

#### OCT, OCTA and VFI: patient–control comparison

Optical coherence tomography (OCT) parameters were evaluated using quadrant-based analyses in both the retina and macular regions. In the retinal region average RNFL and in the macular region, perifoveal inferior quadrant, parafoveal superior, nasal, inferior, and temporal quadrants, were significantly thinner in the severe OSAS group compared with controls (p = 0.024, p = 0.001, p = 0.037, p = 0.022, p = 0.002 and p = 0.004, respectively). Although ganglion cell layer (GCL) mean and minimum thickness values, were thinner in the patient group than in controls, the difference did not reach statistical significance (p > 0.05). Furthermore, optic disc parameters, including C/D, rim area, and disc area, did not differ significantly between the groups (p > 0.05). After adjustment for BMI using ANCOVA, perifoveal inferior macular thickness parameters remained significant, whereas the difference in average RNFL thickness no longer reached statistical significance. However, after subsequent FDR correction within parameter families, these differences did not remain statistically significant. Therefore, baseline between-group findings were interpreted cautiously as exploratory observations, possibly influenced by interindividual variability and BMI-related confounding.

When optical coherence tomography angiography (OCTA) parameters were compared, no significant differences were observed between groups (p > 0.05).

For visual field analyses, the eye with the worse mean deviation (MD) value was selected for statistical evaluation. Compared with controls, patients with severe OSAS demonstrated significantly worse visual field indices (p = 0.047). Intraocular pressure (IOP) values were similar between the two groups (p = 0.816). OCT, OCTA and VFI quadrant analyses are summarized in Table 2.

**Table 2:** Comparison of OCT, OCTA, and visual field parameters between the severe OSAS and control groups

Variable	Severe OSAS (N = 38)	Control (N = 19)	p	Adjusted p ANCOVA	FDR p	Partial η^2^
	Mean ± SD	Mean ± SD				
**OCT-retina parameters**						
*PP-temporal*	62.14 ± 11.78	64.23 ± 12.5	0.813	0,531	0.920	.007
*PP-nazal*	61 ± 7.11	63.59 ± 9.48	0.325	0.343	0.918	.017
*PP-superior*	109 ± 29.26	110.97 ± 15.18	0.366	0.666	0.920	.003
*PP-inferior*	116.97 ± 13.29	117.78 ± 21.62	0.434	0.773	0.920	.002
*RNFL thickness*	82.07 ± 12.25	89.47 ± 9.22	**0.024**	0.059	0.413	.065
*C/D*	0.46 ± 0.15	0.44 ± 0.13	0.362	0.920	0.920	.001
*Rim area*	1.47 ± 0.32	1.51 ± 0.43	0.886	0.787	0.920	.001
**OCT-macula parameters**						
*M-perifoveal-süperior*	279.58 ± 12.79	286.15 ± 22.88	0.117	0.133	0.276	.041
*M-perifoveal-nazal*	295.34 ± 14.32	301.69 ± 17.07	0.062	0.158	0.276	.037
*M-perifoveal-inferior*	265.16 ± 12.26	275.49 ± 15.78	**0.001**	**0.046**	0.250	.072
*M-perifoveal-temporal*	261.92 ± 12.07	270.37 ± 14.82	0.066	0,138	0.276	.048
*M-parafoveal-superior*	321.92 ± 15.37	329.97 ± 20.49	**0.037**	0.175	0.250	.034
*M-parafoveal-nazal*	322.5 ± 14.01	332.84 ± 21.23	**0.022**	0.131	0.276	.042
*M-parafoveal-inferior*	317.13 ± 14.23	330.08 ± 19.81	**0.002**	0.318	0.398	.018
*M-parafoveal-temporal*	307.95 ± 11.94	318.66 ± 20.65	**0.004**	0.129	0.276	.042
*Central foveal thickness*	253.61 ± 21.48	254.95 ± 49.22	0.906	0.812	0.812	.001
*Macular volume (mm*^*3*^*)*	9.91 ± 0.42	10.09 ± 1	0.322	0.432	0.480	.011
**Ganglion cell layer**						
*GCL-mean*	79.73 ± 6.18	81.22 ± 7.28	0.638	0.654	1.000	.001
*GCL-minimum*	63.92 ± 26.95	66.26 ± 22.63	0.972	0.930	0.930	<.001
**OCTA parameters**						
SCP_VD_Whole	50.79 ± 2.73	50.56 ± 2.59	0.090	0.793	0.952	.001
DCP_VD_Whole	52.13 ± 4.86	52.74 ± 5.23	0.060	0.595	0.893	.005
FAZ_area	0.28 ± 0.09	0.26 ± 0.1	0.651	0.981	0.981	<.001
FAZ perimeter	2.01 ± 0.3	1.95 ± 0.41	0.772	0.572	0.893	.008
FAZ FD	55.42 ± 3.62	53.83 ± 3.68	0.580	0.265	0.795	.023
**Optic nerve head OCTA**						
RPC (Whole, Small Vessels)	52.51 ± 2.4	52 ± 2.68	0.846	0.198	0.795	.030
**Visual field parameters**						
VFI 24–2	96.17 ± 3.3	93.99 ± 7.8	**0.047**	**0.016**	0.080	.103
MD 24–2	−3.68 ± 3.43	−3.02 ± 2.08	0.390	0.781	0.910	.001
PSD 24–2	4.07 ± 2.33	3.35 ± 2.84	0.210	0.451	0.910	.011
MD 10–2	−2.85 ± 2.9	−2.67 ± 2.14	0.550	0.910	0.910	<.001
PSD 10–2	1.77 ± 1.25	0.94 ± 2.08	0.720	0.851	0.910	.001

OSAS, obstructive sleep apnea syndrome; OCT, optical coherence tomography; OCTA, optical coherence tomography angiography; PP, peripapillary; RNFL, retinal nerve fiber layer; C/D, cup-to-disc ratio; GCL, ganglion cell layer; SCP, superficial capillary plexus; DCP, deep capillary plexus; VD, vessel density; FAZ, foveal avascular zone; FD, fractal dimension; RPC, radial peripapillary capillary; IOP, intraocular pressure; VF, visual field; VFI, visual field index; MD, mean deviation; PSD, pattern standard deviation. Raw p values were obtained using independent-samples Student’s t-tests. Adjusted p values were calculated using ANCOVA with BMI as a covariate. FDR-adjusted p values were calculated using the Benjamini–Hochberg procedure within each parameter family. Partial eta squared values are presented as measures of effect size

### Pre-treatment vs post-treatment comparisons

#### Changes in PSG and ESS during follow-up

Among the 38 patients who initiated CPAP treatment, 8 were unable to use the device in accordance with the predefined adherence criteria during the 6-month follow-up period and were therefore excluded from the pre–post analyses. Consequently, treatment-related comparisons were performed in 30 patients.

After 6 months of CPAP therapy, ESS scores and all PSG parameters showed statistically significant improvement compared with baseline (all p < 0.05; Table 3). All PSG-related parameters and ESS scores remained statistically significant after FDR correction.

**Table 3:** Comparison of polysomnography parameters and visual field indices before and after treatment in patients with severe OSAS

Variable	Pre-treatment (N = 30)	Post-treatment (N = 30)	Raw p	FDR p
	Mean ± SD	Mean ± SD		
ESS	8.8 ± 5.6	6.2 ± 4.95	**0.025**	**0.025**
**PSG parameters**				
AHI	50.7 ± 20.5	0.8 ± 1.16	** < 0.001**	** < 0.001**
Lsat	76.1 ± 9.9	88.2 ± 2.6	** < 0.001**	** < 0.001**
mO_2_	91.7 ± 3.6	95.01 ± 0.9	** < 0.001**	** < 0.001**
Tsat	22.7 ± 23.5	0.21 ± 0.27	** < 0.001**	** < 0.001**

PSG, polysomnography; ESS, Epworth Sleepiness Scale; AHI, apnea–hypopnea index; mO_2_, mean oxygen saturation; Lsat, lowest oxygen saturation; Tsat, time with low oxygen saturation. Raw p values were obtained using paired-samples t-tests. FDR-adjusted p values were calculated using the Benjamini–Hochberg procedure within each parameter family

### OCT: pre-treatment vs post-treatment

In OCT comparisons, RNFL thickness, peripapillary inferior significantly increased after treatment (p = 0.003, p = 0.009 respectively), whereas no significant change was observed in ganglion cell layer (GCL) thickness (p = 0.770). Sub-analyses demonstrated a significant increase in cup-to-disc ratio (C/D) and rim area after CPAP (p = 0.001, p < 0.001 respectively). In the macular region, perifoveal temporal and parafoveal temporal quadrants showed significant thickening after treatment (p = 0.030 and p = 0.014, respectively), while no significant changes were observed in the remaining quadrants. After correction for multiple comparisons using the false discovery rate (FDR) method, the increase in average RNFL thickness, inferior peripapillary RNFL thickness, C/D ratio, and reduction in rim area remained statistically significant. Although perifoveal and parafoveal temporal macular thicknesses demonstrated nominal significance in uncorrected analyses, these associations did not remain significant after FDR correction. OCT and quadrant analyses are summarized in Table 4.

**Table 4:** Comparison of OCT, OCTA, and visual field parameters before and after treatment in patients with severe OSAS

Variable	Pre-treatment (N = 30)	Post-treatment (N = 30)	Raw p	FDR p
	Mean ± SD	Mean ± SD		
**OCT-retina parameters**				
PP-temporal	59.50 ± 10.95	62.13 ± 14.14	0.150	0.175
PP-nazal	61.20 ± 10.78	63.60 ± 6.86	0.145	0.175
PP-superior	107.43 ± 19.83	109 ± 17.07	0.603	0.603
PP-inferior	111.40 ± 16.18	117.8 ± 17.8	**0.009**	**0.016**
Average RNFL thickness	81.90 ± 13.46	85.23 ± 13	**0.003**	**0.010**
C/D	0.43 ± 0.16	0.52 ± 0.12	**0.001**	**0.007**
Rim area	1.52 ± 0.36	1.34 ± 0.29	** < 0.001**	** < 0.001**
**OCT-macula parameters**				
M-perifoveal-süperior	277.7 ± 15.6	278.60 ± 16.62	0.738	0.987
M-perifoveal-nazal	297.1 ± 18.32	299.76 ± 21.64	0.458	0.917
M-perifoveal-inferior	271.2 ± 14.41	275.3 ± 18.84	0.146	0.365
M-perifoveal-temporal	264.1 ± 18.16	271.33 ± 26.66	**0.030**	0.101
M-parafoveal-superior	323.83 ± 20.55	324.3 ± 39.3	0.949	0.987
M-parafoveal-nazal	325.6 ± 24.09	326.76 ± 19.35	0.785	0.981
M-parafoveal-inferior	327.76 ± 22.77	327.8 ± 20.23	0.987	0.987
M-parafoveal-temporal	311.3 ± 22.75	317.03 ± 20.03	**0.014**	0.072
Central foveal thickness	247.93 + − 39.21	250.1 ± 38.82	0.599	0.981
Macular volume (mm^3^)	10.29 ± 1.94	10.12 ± 0.78	0.634	0.981
**Ganglion cell layer**				
GCL-mean	76.26 ± 17.70	76.73 ± 16.32	0.770	0.770
GCL-minimum	67.26 ± 20.16	68.70 ± 19.11	0.116	0.232
**OCTA parameters**				
SCP_VD_Whole	50.21 ± 2.55	49.59 ± 4.56	0.152	0.304
DCP_VD_Whole	51.74 ± 5.09	49.23 ± 4.56	**0.030**	0.180
FAZ_area	0.26 ± 0.09	0.27 ± 0.08	0.698	0.912
FAZ perimeter	1.97 ± 0.42	2.02 ± 0.35	0.596	0.912
FAZ FD	53.97 ± 3.66	53.82 ± 2.58	0.797	0.912
**Optic nerve head OCTA**				
RPC whole small vessels	51.32 ± 2.57	51.18 ± 6.22	0.912	0.912
**Visual field parameters**				
VFI 24–2	98.14 ± 1.54	92.19 ± 13.49	**0.019**	**0.025**
MD 24–2	− 2.55 ± 3.71	−0.88 ± 2.02	**0.017**	**0.025**
PSD 24	4.42 ± 2.76	3.26 ± 1.36	**0.020**	**0.025**
MD 10	− 2.27 ± 3.16	−1.89 ± 1.15	0.512	0.512
PSD 10	1.83 ± 1.40	0.97 ± 0.50	**0.003**	**0.015**

OSAS, obstructive sleep apnea syndrome; OCT, optical coherence tomography; OCTA, optical coherence tomography angiography; PP, peripapillary; RNFL, retinal nerve fiber layer; C/D, cup-to-disc ratio; GCL, ganglion cell layer; SCP, superficial capillary plexus; DCP, deep capillary plexus; VD, vessel density; FAZ, foveal avascular zone; FD, fractal dimension; RPC, radial peripapillary capillary; IOP, intraocular pressure; VF, visual field; VFI, visual field index; MD, mean deviation; PSD, pattern standard deviation. Raw p values were obtained using paired-samples t-tests. FDR-adjusted p values were calculated using the Benjamini–Hochberg procedure within each parameter family

### OCTA: pre-treatment vs post-treatment

After 6 months of CPAP treatment, OCTA analysis demonstrated a significant reduction in deep capillary plexus (DCP) vessel density (p = 0.030). Although a reduction trend in deep capillary plexus (DCP) vessel density was observed after CPAP therapy in uncorrected analyses, this association did not remain statistically significant after FDR correction. No significant pre–post differences were observed in the remaining OCTA parameters (Table 4).

### Visual field: pre-treatment vs post-treatment

In the 24–2 visual field assessment (retinal evaluation), VFI, MD and PSD values significantly improved after CPAP treatment compared with baseline (p = 0.019, p = 0.017, and p = 0.020, respectively). Improvements in VFI, MD, and PSD in the 24–2 visual field assessment, as well as PSD in the 10–2 test, remained statistically significant after FDR correction.

In the 10–2 macular evaluation, MD tended to improve after treatment, although the difference did not reach statistical significance (p = 0.512), whereas PSD was significantly lower following CPAP treatment (p = 0.003; Table 4).

### Correlation analyses

**Fig. 1:**
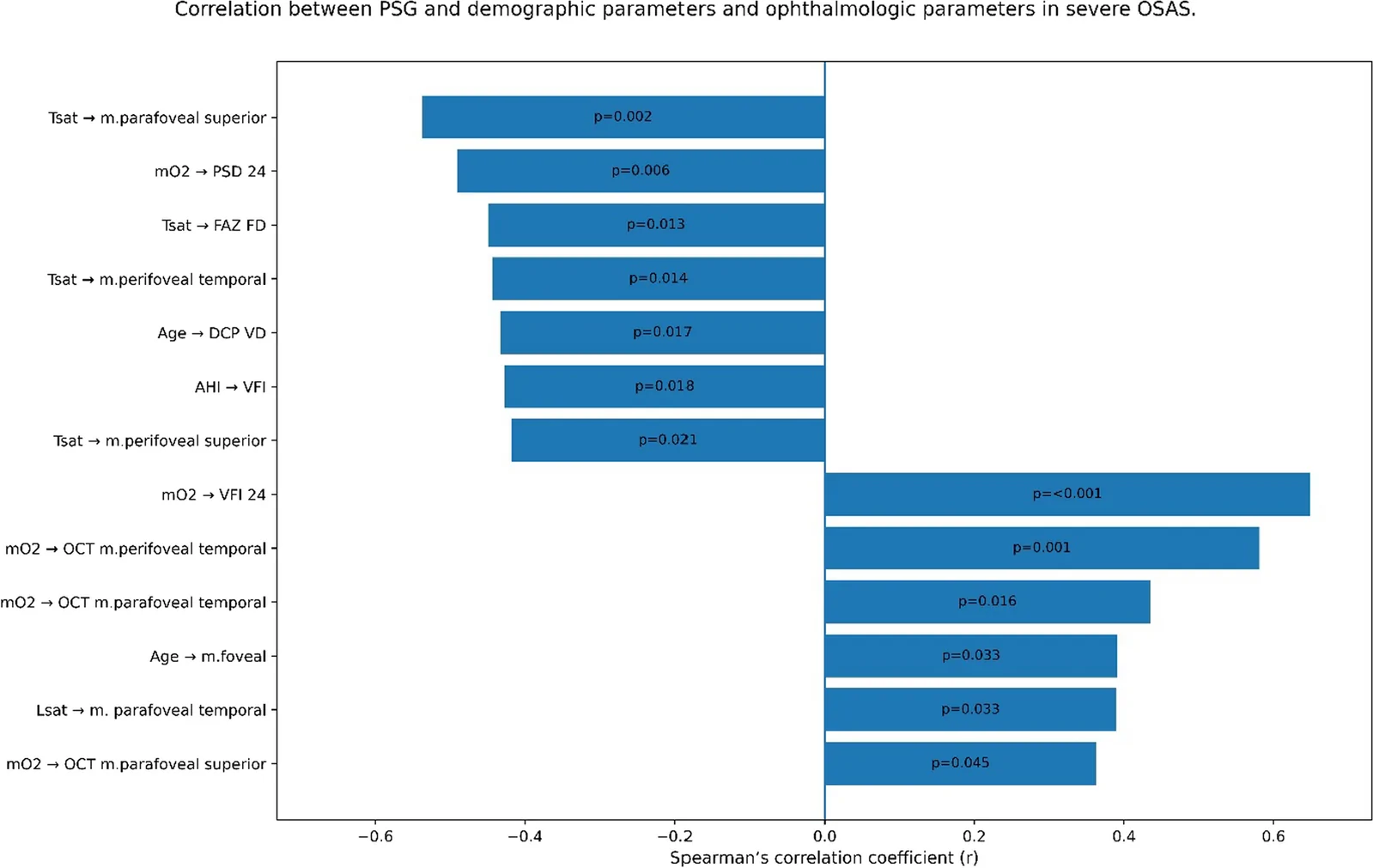
Significant correlations between PSG/demographic variables and ophthalmologic parameters in severe OSAS. Before treatment. Cells display Spearman’s correlation coefficients (r). Only statistically significant correlations (p < 0.05) are shown. AHI: apnea–hypopnea index; SpO_2_: peripheral oxygen saturation; mO_2_: mean oxygen saturation; Lsat: lowest oxygen saturation; Tsat: cumulative time spent with oxygen saturation < 90%; OCT: optic coherent tomography, m: macular, DCP: deep capillary plexus; FD: flow density; VD: vessel density; FAZ: foveal avascular zone; VFI: visual field index; PSD: pattern standard deviation; MD: mean deviation

**Fig. 2:**
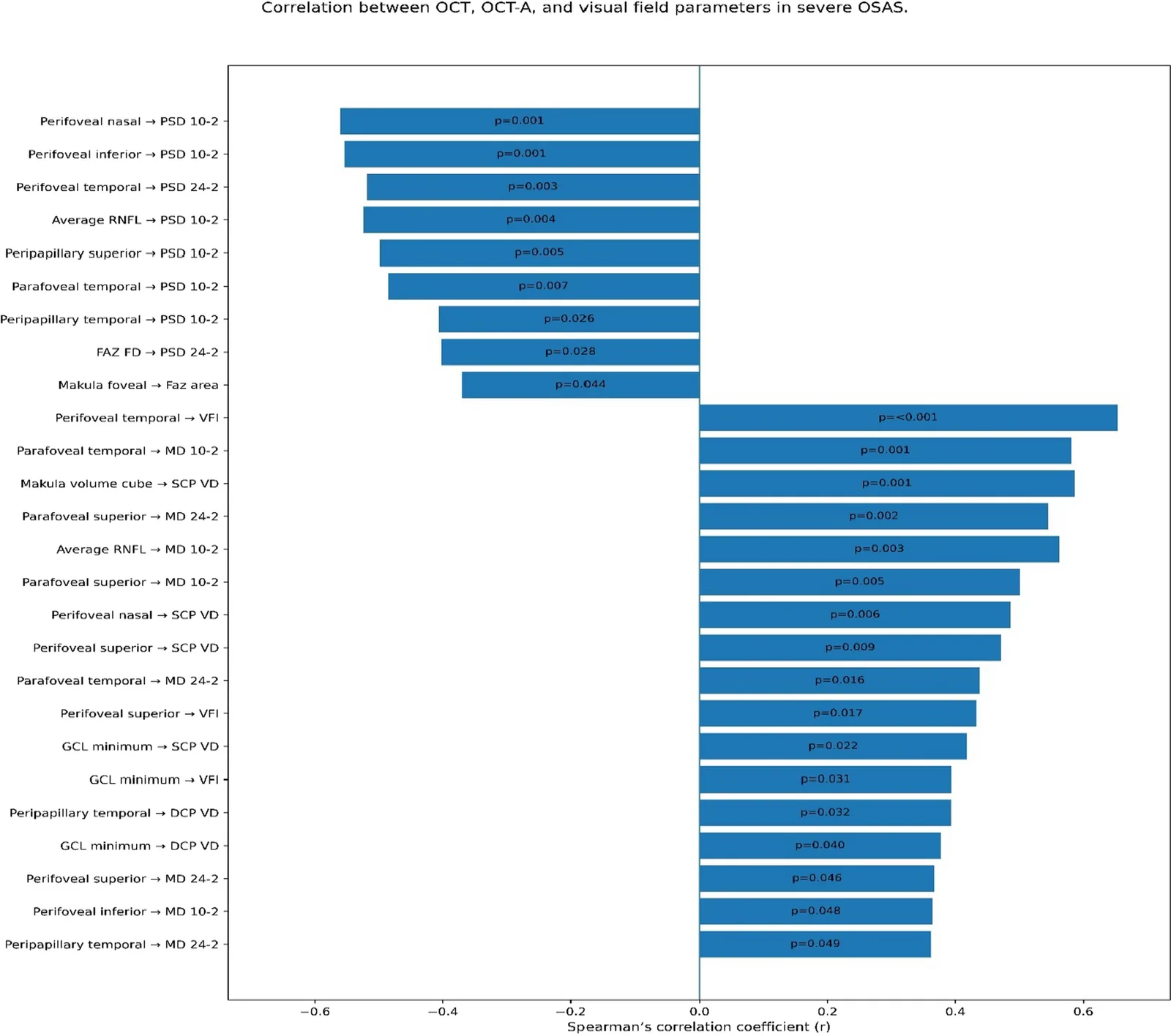
Significant correlations between OCT structural parameters, OCT-A microvascular metrics, and visual field indices in severe OSAS. Bars display Spearman’s correlation coefficients (r). Exact p-values are shown within each bar. Only statistically significant correlations (p < 0.05) are presented. OCT: optical coherence tomography; OCT-A: optical coherence tomography angiography; RNFL: retinal nerve fiber layer SCP: superficial capillary plexus; DCP: deep capillary plexus; VD: vessel density; MD (24–2/10–2): mean deviation in Humphrey visual field 24–2 and 10–2 protocols; PSD (24–2/10–2): pattern standard deviation in Humphrey visual field 24–2 and 10–2 protocols; VFI: visual field index; GCL: ganglion cell layer; FAZ: foveal avascular zone;

Correlation analyses were performed between polysomnographic parameters and ophthalmological measurements in participants who adhered to CPAP treatment before treatment. OSAS duration demonstrated weak but statistically significant correlations with AHI (r = 0.345, p = 0.034) and ESS scores (r = 0.322, p = 0.047), whereas no significant correlations were observed between OSAS duration and neuro-ophthalmologic parameters. Strong correlations were observed between polysomnographic parameters and ophthalmological measurements as well as inter-ophthalmological measurements. Correlation findings are summarized in Fig. 1 and Fig. 2.

## Discussion

In this prospective case–control study, we evaluated structural, microvascular, and functional neuro-ophthalmologic parameters in participants meeting predefined inclusion and exclusion criteria using OCT, OCTA, and visual field testing (retinal and macular), and investigated the effects of 6-month CPAP therapy in patients with severe OSAS. In baseline patient–control comparisons, several OCT-derived macular and visual field parameters demonstrated nominal differences between groups; however, these associations did not remain statistically significant after BMI adjustment and FDR correction. In contrast, longitudinal within-subject analyses demonstrated significant improvements in selected RNFL and visual field parameters following CPAP therapy, together with robust improvement in PSG and ESS measures after FDR correction. Although a reduction trend in DCP vessel density was observed after treatment, this association did not remain significant after multiple-comparison correction. Taken together, these findings suggest that longitudinal multimodal assessment may provide a more robust framework for evaluating treatment-related neuro-ophthalmologic changes in severe OSAS. To date, no study has simultaneously evaluated OCT, OCTA, and both retinal (24–2) and macular (10–2) visual field outcomes before and after CPAP therapy in the same severe OSAS cohort. Existing studies generally focus on a single modality or cross-sectional comparisons. Therefore, our study provides a comprehensive multimodal assessment integrating structural, vascular, and functional biomarkers into a treatment-response framework.

The effects of obstructive sleep apnea syndrome on the optic nerve and retinal structures are thought to involve multiple interacting mechanisms, including intermittent hypoxia, sympathetic activation, inflammation, endothelial dysfunction, oxidative stress, and microangiopathy, all of which may impair optic nerve head perfusion [7, 9]. Additional factors such as nocturnal systemic hypotension, pronounced nocturnal blood pressure dipping, vasospasm, and increased blood viscosity have also been proposed to increase vascular resistance in the small vessels supplying the optic nerve, thereby predisposing to transient or permanent ischemic injury [10, 15]. Together, these processes provide a potential pathophysiological basis for OSAS -related axonal dysfunction followed by structural and functional impairment.

Several studies have reported RNFL thinning in patients with moderate-to-severe OSAS, with greater thinning observed as disease severity increases [16, 17]. Proposed mechanisms include hypoxia-related perfusion instability and vasospasm leading to axonal dysfunction and loss [18–20]. Studies focusing on CPAP therapy have described early improvements in peripapillary RNFL thickness and increases in choroidal thickness [21, 22]. A report suggests that RNFL improvement may emerge within the first months of treatment and subsequently plateau, with limited additional change between months 3 and 12 [23]. In our study, mean RNFL thickness was numerically lower in the OSAS group than in controls, however this difference did not remain statistically significant after BMI adjustment and FDR correction. In contrast, after 6 months of effective CPAP therapy, a significant increase in average and inferior peripapillary RNFL thickness remained significant after FDR correction. Although nominal thickening trends were observed in selected macular regions following treatment, these associations did not remain significant after correction for multiple comparisons. These findings support the hypothesis that a component of OSAS-related axonal dysfunction may be at least partially reversible with effective treatment of nocturnal hypoxia and sleep fragmentation.

Recent studies indicate that macular structural measurements in individuals with OSAS may differ from those in healthy controls. Indeed, several series have reported macular thinning, particularly in moderate-to-severe OSAS, attributed to intermittent hypoxia–related retinal neurodegeneration [16, 18]. In contrast, other studies have found preserved macular thickness or variable segmental patterns [24]. In our baseline patient–control comparison, several macular parameters were nominally thinner in the OSAS group, consistent with the literature. Following 6 months of effective CPAP therapy, a general trend toward increased macular thickness was observed; however, statistical significance was limited to the perifoveal and parafoveal temporal segments. However, these associations observed after CPAP therapy should be interpreted cautiously, as these findings did not remain significant after correction for multiple comparisons Notably, GCL thickness did not change significantly between patient and control groups or after CPAP therapy in severe OSAS. This finding suggests that CPAP may primarily ameliorate axonal dysfunction at the RNFL level and components related to subclinical edema or metabolic stress, whereas neuronal cell bodies may either be relatively preserved at baseline or have limited short-term reversibility. The variability of GCL findings in the OSAS literature—ranging from thinning to no change or even paradoxical thickening—also indicates that ganglion cell involvement may depend on disease severity, duration of hypoxia, and timing of assessment [24–28].

The retina is one of the tissues with the highest blood flow per gram in the human body and is considered particularly sensitive to hypoxia-related microvascular dysregulation [29]. OCTA provides noninvasive quantitative assessment of retinal microvascular circulation, including the superficial capillary plexus (SCP), deep capillary plexus (DCP), and foveal avascular zone (FAZ). SCP and DCP measurements may reflect different aspects of retinal perfusion and autoregulatory response, whereas FAZ-related metrics are generally considered markers of macular microvascular integrity. Previous OCTA studies in OSAS have reported heterogeneous findings, including both reduced and increased vessel density values, possibly reflecting differences in disease severity, hypoxic burden, and compensatory vascular mechanisms [30]. Although OCTA studies in OSAS have reported reduced vessel density in the superficial and deep capillary plexuses, findings remain inconsistent [31, 32]. Interestingly, some studies suggest that chronic hypoxia may trigger compensatory vasodilation and collateral recruitment, potentially resulting in increased DCP metrics in certain patient groups, consistent with a retinal autoregulatory response [33].

In our baseline comparisons, no OCTA parameter remained statistically significant after BMI adjustment and FDR correction. The observed reduction in DCP vessel density after treatment should be interpreted cautiously, as this finding did not remain statistically significant after correction for multiple comparisons. Measurement variability, segmentation-related factors, and physiological fluctuations inherent to OCTA imaging may also contribute to this observation.

Obstructive sleep apnea syndrome is a recognized risk factor for glaucomatous optic neuropathy and non-arteritic anterior ischemic optic neuropathy [8, 34]. CPAP therapy may also influence ocular physiology through mechanisms involving episcleral venous pressure, cerebrospinal fluid dynamics, and translaminar pressure gradients. Although an increase in the C/D ratio was observed after CPAP, the concurrent increase in RNFL thickness and improvement in visual field indices argue against progressive glaucomatous damage. Therefore, the change in C/D ratio may reflect CPAP-related biomechanical or pressure-mediated alterations at the optic nerve head rather than true glaucomatous progression [9, 35, 36].

The impact of OSAS on visual field metrics remains controversial. Some studies have reported more pronounced visual field loss or glaucomatous-like optic disc changes in moderate-to-severe OSAS compared with normotensive controls, whereas others have found no significant differences [16, 18, 37, 38]. In our study, baseline visual field differences between OSAS patients and controls did not remain statistically significant after BMI adjustment and FDR correction More importantly, within-patient comparisons demonstrated significant improvement in VFI, MD, and PSD on the 24–2 test after CPAP therapy and these associations remained significant after FDR correction. In contrast, in the macular 10–2 test, MD remained unchanged while PSD decreased significantly after treatment. This dissociation between MD and PSD may be clinically meaningful, as MD primarily reflects diffuse/global sensitivity loss, whereas PSD captures localized functional irregularities that may be more responsive to improvements in nocturnal hypoxic burden and perfusion instability. The coexistence of RNFL improvement and visual field recovery further supports the concept that at least part of OSAS-related neuro-ophthalmologic dysfunction may reflect reversible hypoxia-related neuronal stress rather than irreversible axonal loss. Given that central retinal ganglion cell involvement has been described in OSAS and may preferentially affect the macular region [26], functional improvement in macular perimetry may precede detectable structural recovery on OCT, particularly during short-term follow-up.

After BMI adjustment and FDR correction, the baseline patient–control differences in neuro-ophthalmologic parameters were no longer statistically significant, suggesting that these cross-sectional findings should be interpreted cautiously. This may reflect the modest sample size, the influence of BMI-related confounding, and the conservative nature of multiple-comparison correction across numerous ophthalmologic parameters. Importantly, however, the within-subject pre–post CPAP design represents a more robust framework for evaluating treatment-related change, as each patient served as his or her own control. The persistence of significant changes in selected OCT-retina and visual field parameters after FDR correction therefore strengthens the evidence that effective CPAP therapy may be associated with measurable structural and functional neuro-ophthalmologic improvement in severe OSAS.

Recent studies indicate that hypoxic burden and disease severity measured by PSG may correlate with retinal structural and microvascular parameters; however, the direction and strength of these correlations—particularly for OCTA vessel density and visual field measures—remain heterogeneous across studies [17, 33, 39, 40]. In our correlation analysis (Fig. 1), increasing oxygenation impairment was associated with a trend toward thinning of OCT-measured retinal and macular layers. Similarly, higher hypoxic burden was associated with increased PSD values and more negative MD values. Correlations between structural OCT parameters and OCTA and visual field measures (Fig. 2) demonstrated that peripapillary and macular thickness metrics were significantly associated with functional visual field indices and capillary plexus densities. This pattern suggests that neurodegenerative and microvascular processes in OSAS are interrelated but may not progress in a strictly parallel manner. Therefore, a multimodal assessment approach may facilitate a more comprehensive characterization of OSAS-related neuro-ophthalmologic alterations. In the present cohort, most patients reported symptom onset within the previous year, resulting in a relatively homogeneous early-stage severe OSAS population. OSAS duration demonstrated significant correlations with AHI and ESS scores, suggesting an association between symptom duration and clinical disease severity. However, no significant correlations were observed between reported symptom duration and neuro-ophthalmologic parameters. This finding should be interpreted cautiously, as patient-reported symptom duration may not accurately reflect the true duration of OSAS and showed limited variability within our cohort.

This study has several limitations. First, the sample size was moderate, and the number of patients available for paired pre- and post-treatment comparisons decreased due to suboptimal CPAP adherence, which may limit statistical power and generalizability. Second, OCTA measurements are sensitive to image quality, segmentation errors, and motion artifacts; despite standardized acquisition protocols, vessel density measurements may have been affected by these factors. Third, given the relatively high number of correlation analyses and the moderate sample size, some retinal associations may have been underpowered, and the possibility of type II error cannot be excluded. In addition, although BMI-adjusted analyses were performed, residual confounding related to obesity cannot be completely excluded. Finally, additional subgroup analyses based on symptom burden or other clinical characteristics were not feasible because of the relatively limited sample size.

In conclusion, effective CPAP therapy was associated with robust structural and functional improvement in severe OSAS, particularly at the RNFL and visual field level. Larger longitudinal studies with longer follow-up are needed to better clarify the temporal relationship among structural recovery, vascular remodeling, and functional improvement in OSAS.

## Data Availability

The datasets generated and/or analyzed during the current study are not publicly available due to patient privacy and institutional regulations but are available from the corresponding author on reasonable request.

## References

[1] Ho ML, Brass SD (2011) Obstructive sleep apnea. Neurol Int 3(3):e15. 10.4081/ni.2011.e15 PMC3286155 22368774

[2] Patel SR (2019) Obstructive sleep apnea. Ann Intern Med 171(11):ITC81–ITC96. 10.7326/AITC201912030 31791057

[3] Gottlieb DJ, Punjabi NM (2020) Diagnosis and management of obstructive sleep apnea: a review. JAMA 323(14):1389–1400. 10.1001/jama.2020.3514 32286648

[4] Malhotra A, Ayappa I, Ayas N, Collop N, Kirsch D, McArdle N, Mehra R, Pack AI, Punjabi N, White DP, Gottlieb DJ (2021) Metrics of sleep apnea severity: beyond the apnea-hypopnea index. Sleep 44(7):zsab030. 10.1093/sleep/zsab030 PMC8271129 33693939

[5] Tingting X, Danming Y, Xin C (2018) Non-surgical treatment of obstructive sleep apnea syndrome. Eur Arch Otorhinolaryngol 275(2):335–346. 10.1007/s00405-017-4818-y 29177626

[6] de Terán TD, Boira I, Cerveró A, Casado A, Lopez-de-Eguileta A, Fonseca S, Muñoz P, Nebot C, Nicolini A, Banfi P, Solidoro P, González M (2025) Benefit of continuous positive airway pressure on optic nerve damage in patients with obstructive sleep apnea. Sleep Breath 29(2):173. 10.1007/s11325-025-03336-w PMC12043755 40304843

[7] Wong B, Fraser CL (2019) Obstructive sleep apnea in neuro-ophthalmology. J Neuroophthalmol 39(3):370–379. 10.1097/WNO.0000000000000728 30300256

[8] Fraser CL (2019) Update on obstructive sleep apnea for neuro-ophthalmology. Curr Opin Neurol 32(1):124–130. 10.1097/WCO.0000000000000630 30418196

[9] Batum M, Kısabay A, Mayalı H, Göktalay T, Kurt E, Selçuki D, Yılmaz H (2020) Evaluation of effects of positive airway pressure treatment on retinal fiber thickness and visual pathways using optic coherence tomography and visual evoked potentials in the patients with severe obstructive sleep apnea syndrome. Int Ophthalmol 40(10):2475–2485. 10.1007/s10792-020-01426-0 32506292

[10] Hayreh SS (2009) Ischemic optic neuropathy. Prog Retin Eye Res 28(1):34–62. 10.1016/j.preteyeres.2008.11.002 19063989

[11] Lee SSY, Nilagiri VK, Mackey DA (2022) Sleep and eye disease: a review. Clin Exp Ophthalmol 50(3):334–344. 10.1111/ceo.14071 PMC9544516 35263016

[12] Liu PK, Chiu TY, Wang NK, Levi SR, Tsai MJ (2021) Ocular complications of obstructive sleep apnea. J Clin Med 10(15):3422. 10.3390/jcm10153422 PMC8348497 34362205

[13] Johns MW (1991) A new method for measuring daytime sleepiness: The Epworth Sleepiness Scale. Sleep 14(6):540–545. 10.1093/sleep/14.6.540 1798888

[14] Ağargün MY, Çilli AS, Kara H, Bilici M, Telcioğlu M, Semiz ÜB (1999) Epworth uykululuk ölçeğinin geçerliliği ve güvenilirliği. Turk Psikiyatri Derg 10(4):261–267

[15] Hayreh SS, Zimmerman MB (2008) Nonarteritic anterior ischemic optic neuropathy: natural history of visual outcome. Ophthalmology 115(2):298-305.e2. 10.1016/j.ophtha.2007.05.031 PMC2782939 17698200

[16] Casas P, Ascaso FJ, Vicente E et al (2018) Visual field defects and retinal nerve fiber imaging in patients with obstructive sleep apnea syndrome and in healthy controls. BMC Ophthalmol 18:66. 10.1186/s12886-018-0728-z PMC5833149 29499674

[17] Davanian A, Williamson L, Taylor C et al (2022) Optical coherence tomography angiography and Humphrey visual field in patients with obstructive sleep apnea. J Clin Sleep Med 18(9):2133–2142. 10.5664/jcsm.10054 PMC9435350 35532117

[18] Lin PW, Lin HC, Friedman M, Chang HW, Salapatas AM, Lin MC, Chen YC (2020) Effects of CPAP for patients with OSA on visual sensitivity and retinal thickness. Sleep Med 67:156–163. 10.1016/j.sleep.2019.10.019 31927222

[19] Sagiv O, Mathalone N, Kassem R, Koudsi N, Assia EI (2014) Retinal nerve fiber layer thickness measurement in patients with obstructive sleep apnea syndrome. Clin Exp Ophthalmol 42(2):132–137. 10.1111/ceo.12123 23777553

[20] Casas P, Ascaso FJ, Cristóbal JA (2013) Effect of obstructive sleep apnea syndrome on retinal nerve fiber layer thickness. Acta Ophthalmol 91(7):e505–e510. 10.1111/aos.12153

[21] Santos M, Hofmann RJ, Cunha JP, Alves M, Viana P, Cunha-Vaz J (2017) Choroidal thickness in obstructive sleep apnea syndrome before and after continuous positive airway pressure treatment. Retina 37(12):2371–2378

[22] Christou E, Georgalas I, Papaconstantinou D, Ladas I, Chatziralli I (2022) Effect of continuous positive airway pressure treatment on retinal nerve fiber layer thickness in patients with obstructive sleep apnea syndrome. Graefes Arch Clin Exp Ophthalmol 260(3):843–850. 10.1007/s00417-021-05455-6

[23] Naranjo-Bonilla P, Giménez-Gómez R, Muñoz-Villanueva MDC, Jurado-Gámez B (2022) Retinal and choroidal effects of continuous positive airway pressure as treatment for sleep apnea: results at 12 months. Int J Environ Res Public Health 19(19):12637. 10.3390/ijerph191912637 PMC9566654 36231937

[24] Moyal L, Blumen-Ohana E, Blumen M et al (2018) Parafoveal and optic disc vessel density in patients with obstructive sleep apnea syndrome: An OCT angiography study. Graefe’s Arch Clin Exp Ophthalmol 256(7):1235–1243. 10.1007/s00417-018-3943-7 29577172

[25] Huseyinoglu N, Ekinci M, Ozben S et al (2014) Optic disc and retinal nerve fiber layer parameters as indicators of neurodegenerative brain changes in patients with obstructive sleep apnea syndrome. Sleep Breath 18(1):95–102. 10.1007/s11325-013-0854-z 23636560

[26] Kara N, Sayin N, Bayramoglu SE, Savas AU (2018) Peripapillary retinal nerve fiber layer thickness and macular ganglion cell layer thickness in patients with obstructive sleep apnea syndrome. Eye 32(4):701–706. 10.1038/eye.2017.279 PMC5898860 29271421

[27] Chalkiadaki E, Andreanos K, Karmiris E et al (2021) Ganglion cell layer thickening in patients suffering from obstructive sleep apnea–hypopnea syndrome with long mean apnea–hypopnea duration during sleep. Int Ophthalmol 41:923–935. 10.1007/s10792-020-01648-2 33201446

[28] Sundaram V, Haridas S, Dhar M, Harikrishnan CP (2022) Evaluation of retinal nerve fiber layer, ganglion cell layer, and optic nerve head morphological parameters in patients with obstructive sleep apnea and comparison with normal population. Indian J Ophthalmol 70(1):453–457. 10.4103/ijo.IJO_985_21 PMC9023914 35086215

[29] Choi W, Mohler KJ, Potsaid B et al (2013) Choriocapillaris and choroidal microvasculature imaging with ultrahigh-speed OCT angiography. PLoS ONE 8(12):e81499. 10.1371/journal.pone.0081499 PMC3859478 24349078

[30] Christou EE, Kostikas K, Asproudis C, Zafeiropoulos P, Stefaniotou M, Asproudis I (2022) Retinal microcirculation characteristics in obstructive sleep apnea/hypopnea syndrome evaluated by OCT-angiography: a literature review. Int Ophthalmol 42(12):3977–3991. 10.1007/s10792-022-02361-y 35604623

[31] Yu J, Xiao K, Huang J, Sun X, Jiang C (2017) Reduced retinal vessel density in obstructive sleep apnea syndrome patients: an OCT angiography study. Invest Ophthalmol Vis Sci 58(9):3506–3512 10.1167/iovs.17-21414 28715584

[32] Ucak T, Unver E (2020) Alterations in parafoveal and optic disc vessel densities in patients with obstructive sleep apnea syndrome. J Ophthalmol 2020:4034382 10.1155/2020/4034382 PMC7056990 32148941

[33] Abdessater L, Hein M, Rasquin F (2024) Analysis of macular vascularization using OCT angiography in patients with obstructive sleep apnea syndrome: a prospective clinical study. Medicina Kaunas 60(5):757. 10.3390/medicina60050757 PMC11122929 38792940

[34] Campbell WW, Barohn JR (2020) The optic nerve. DeJong’s the neurologic examination, 7th ed. Wolters Kluwer, pp 297–370

[35] Uslu H, Kanra AY, Sarac S (2021) Structural assessment of the optic nerve in patients with obstructive sleep apnea syndrome: case-control study. Eur J Ophthalmol 31(4):1741–1748. 10.1177/1120672120926859 32483999

[36] Ulusoy S, Erden M, Dinc ME et al (2015) Effects of use of a continuous positive airway pressure device on glaucoma. Med Sci Monit 21:3415–3419. 10.12659/msm.895897 PMC4642866 26547930

[37] Salzgeber R, Iliev ME, Mathis J (2014) Do optic nerve head and visual field parameters in patients with obstructive sleep apnea syndrome differ from those in control individuals? Klin Monatsbl Augenheilkd 231(4):340–343 10.1055/s-0034-1368260 24771163

[38] Tsang CS, Chong SL, Ho CK, Li MF (2006) Moderate to severe obstructive sleep apnoea patients is associated with a higher incidence of visual field defect. Eye Lond 20(1):38–42. 10.1038/sj.eye.6701785 15650758

[39] Li Y, Liu X, Zhou H et al (2023) Retinal nerve fiber layer thickness in patients with different severities of obstructive sleep apnea. J Glaucoma 32(4):e123–e129. 10.1097/IJG.0000000000002173 36847655

[40] Karabulut M, Bek S, Kutlu G et al (2023) Effects of obstructive sleep apnea on retinal microvasculature. Int J Ophthalmol 16(10):1672–1679. 10.18240/ijo.2023.10.07 PMC10559037 37854376

